# Prevalence of risk factors for noncommunicable diseases in an indigenous community in Santiago Atitlán, Guatemala

**DOI:** 10.26633/RPSP.2017.7

**Published:** 2017-02-08

**Authors:** David Chen, Álvaro Rivera-Andrade, Jessica González, David Burt, Carlos Mendoza-Montano, James Patrie, Max Luna

**Affiliations:** 1 University of Virginia School of Medicine University of Virginia School of Medicine CharlottesvilleVirginia United States of America University of Virginia School of Medicine, Charlottesville, Virginia, United States of America.; 2 Institute of Nutrition of Central America and Panama Institute of Nutrition of Central America and Panama Guatemala City Guatemala Institute of Nutrition of Central America and Panama, Guatemala City, Guatemala.; 3 University of Virginia–Guatemala Initiative University of Virginia–Guatemala Initiative Quetzaltenango Guatemala University of Virginia–Guatemala Initiative, Quetzaltenango, Guatemala.; 4 Department of Emergency Medicine, University of Virginia School of Medicine Department of Emergency Medicine, University of Virginia School of Medicine CharlottesvilleVirginia United States of America Department of Emergency Medicine, University of Virginia School of Medicine, Charlottesville, Virginia, United States of America.; 5 Department of Public Health Sciences University of Virginia School of Medicine CharlottesvilleVirginia United States of America Department of Public Health Sciences, University of Virginia School of Medicine, Charlottesville, Virginia, United States of America.; 6 Division of Cardiovascular Medicine University of Virginia School of Medicine CharlottesvilleVirginia United States of America Division of Cardiovascular Medicine, University of Virginia School of Medicine, Charlottesville, Virginia, United States of America.

**Keywords:** Cardiovascular diseases, metabolic diseases, risk factors, obesity, indigenous population, Guatemala, Enfermedades cardiovasculares, enfermedades metabólicas, factores de riesgo, obesidad, población indígena, Guatemala

## Abstract

**Objective.:**

*To describe the prevalence of noncommunicable disease (NCD) risk factors and assess knowledge of those risk factors in the indigenous community of Santiago Atitlán in Guatemala, a lower-middle income country*.

**Methods.:**

*A population-based, cross-sectional study was conducted using a modified version of the World Health Organization’s STEPS protocol. Adults aged 20–65 years were surveyed regarding demographics and NCD risk factors, and the survey was followed by anthropometric and biochemical measurements*.

**Results.:**

*Out of 501 screened individuals, 350 respondents were enrolled. The mean age was 36.7 years, and 72.3% were women. Over 90% reported earning less than US$ 65 per month. Almost 80% were stunted. Among women, 37.3% were obese and over three-quarters had central obesity. Over three-quarters of the entire group had dyslipidemia and 18.3% had hypertension, but only 3.0% had diabetes. Overall, 36.0% of participants met criteria for metabolic syndrome. There was no significant association between participants’ education and NCD risk factors except for an inverse association with obesity by percent body fat*.

**Conclusions.:**

*Santiago Atitlán is a rural, indigenous Guatemalan community with high rates of poverty and stunting coexisting alongside high rates of obesity, particularly among women. Additionally, high rates of hypertension and dyslipidemia were found, but a low rate of diabetes mellitus. Knowledge of NCDs and their risk factors was low, suggesting that educational interventions may be a high-yield, low-cost approach to combating NCDs in this community*.

## RESUMEN

**Objetivo.:**

Describir la prevalencia de los factores de riesgo de las enfermedades no transmisibles (ENT) y evaluar el conocimiento de esos factores de riesgo en la comunidad indígena de Santiago Atitlán en Guatemala, un país de ingresos medianos bajos.

**Métodos.:**

Se realizó un estudio transversal basado en la población usando una versión modificada del protocolo STEPS de mediciones físicas de la Organización Mundial de la Salud. Se realizó una encuesta a adultos de 20 a 65 años de edad con respecto a algunas características demográficas y los factores de riesgo de las ENT; la encuesta fue seguida de mediciones antropométricas y bioquímicas.

**Resultados.:**

De las 501 personas encuestadas, se registraron 350 para el sondeo. La media de edad fue 36,7 años, y 72,3% eran mujeres. Más de 90% informó tener un ingreso inferior a los US$ 65 mensuales. Casi 80% padecía retraso del crecimiento. Entre las mujeres, 37,3% eran obesas y más de tres cuartas partes tenían obesidad central. Más de tres cuartas partes del grupo tenían dislipidemia y 18,3% tenían hipertensión, pero solo 3,0% tenía diabetes. En términos generales, 36,0% de los participantes satisfacía los criterios de síndrome metabólico. No se observó una asociación significativa entre el nivel de escolaridad de los participantes y los factores de riesgo de las ENT, excepto una asociación inversa con la obesidad por porcentaje de tejidos grasos.

**Conclusiones.:**

Santiago Atitlán es una comunidad indígena rural de Guatemala con tasas elevadas de pobreza y retraso del crecimiento, que coexisten con tasas altas de obesidad, en particular en las mujeres. Además, se encontraron tasas elevadas de hipertensión y dislipidemia, pero una tasa baja de diabetes mellitus. Se observó un conocimiento bajo de las ENT y sus factores de riesgo, lo que indica que las intervenciones educativas pueden ser un enfoque de alto rendimiento y bajo costo para combatir las ENT en esta comunidad.

Public health efforts in developing countries have traditionally focused on infectious diseases and malnutrition. However, there is growing international recognition that the burden of chronic noncommunicable diseases (NCDs), such as diabetes and cardiovascular disease, poses a public health concern in lower-middle income countries (LMICs) (1–2). Globally, over 80% of NCD deaths now occur in LMICs (3). In the Latin America and Caribbean region, NCDs are estimated to be responsible for over half of the disability-adjusted life years lost due to illness (4). Like many other LMICs, Guatemala is undergoing an epidemiologic transition from the predominance of infectious diseases toward the ascendancy of chronic NCDs, largely driven by population aging (5–6), changes in diet, and reductions in physical activity. Previous reports suggest that Guatemala is in an intermediate stage of the transition, where childhood malnutrition and infectious diseases coexist with increasing rates of obesity-related conditions (7–8). In Guatemala, the percentage of deaths attributed to cardiovascular disease (CVD) has increased in recent decades from 7% in 1986 to 17% in 2009, when it represented the second most common cause of death in the country (9). As communities in Guatemala continue to move away from traditional subsistence farming-based lifestyles, diets are transitioning toward the atherogenic and diabetogenic diets seen in most developed countries. As this transition continues, it is expected that NCDs and their risk factors will increase in indigenous communities such as Santiago Atitlán (10).

Santiago is a mixed rural and semi-urban indigenous community in Sololá Department, Guatemala, with a population of approximately 44 000 (11). The majority of the inhabitants speak Tzu’tujil as their native language and Spanish as a second language. Santiago has the highest rate of rural poverty and the lowest literacy rate in the department of Sololá (12), which underscores the importance of assessing NCD knowledge. There is a dearth of quality data on health metrics in Santiago and other indigenous communities, but early reports using convenience samples suggest that NCDs such as hypertension and diabetes are underdiagnosed and undertreated in this rural Mayan community (13–14). Therefore, it is important to study these diseases and their risk factors systematically.

The primary objective of this study was to describe the burden of NCD risk factors in Santiago Atitlán and a secondary objective was to assess community knowledge about NCDs, with the study population in Santiago Atitlán serving as a sample of the indigenous population in Guatemala. Our hypothesis was that this study would confirm the increasing prevalence of NCD risk factors suggested in earlier reports (13–14).

## MATERIALS AND METHODS

### Participants

The Noncommunicable Disease Surveillance (NCDS) Study was designed as a cross-sectional study in a simple random sample to examine the prevalence of behavioral and biochemical NCD risk factors using a modified Spanish version of the World Health Organization’s (WHO) STEPS protocol (15). Application of the Spanish study instruments frequently required verbal translation to Tzu’tujil at the time of the interview since Tzu’tujil is not a written language.

The population of approximately 44 000 was distributed in 20 *cantones* or districts with a total of 7 559 households and an average of 5.8 individuals per household. In order to achieve a geographically representative sample of the population, the number of households to be approached in each *cantón* was determined in proportion to the estimated adult population according to the latest available census data (16). Study households were randomly preselected using household level maps. From October 2012 to April 2013, 501 eligible individuals (1.1% of the total population) were screened for participation in the study. Adults aged 20–65 years who were living in Santiago and were able to provide informed consent were eligible. If a household contained more than one eligible participant, one was randomly selected. Sampling weights were generated to reflect the probability of selection at each stage.

### Study tools

The WHO STEPS protocol is a standardized tool designed for assessing NCD risk factors in developing countries. The original STEPS protocol was modified to be culturally appropriate, to include measurement of body fat percentage (BFP), and to add nine multiple choice questions pertaining to basic knowledge of NCDs (survey instrument available upon request). A pilot phase was conducted in which the study tools were administered to small focus groups drawn from the community to identify potential areas of confusion and to enhance cultural appropriateness. The STEPS protocol is described in detail elsewhere (15). In brief, our instruments included the following:

#### Step 1: Sociodemographic and behavioral variables.

A structured interview based on a standardized questionnaire was used to collect demographic information and data on risk factors. Topics included diet, exercise, smoking, alcohol use, family history, and personal history of NCDs.

#### Step 2: Anthropometric measurements.

Height, weight, waist circumference, BFP, and blood pressure were measured using standardized instruments and protocols. After the subject had been at rest for 5 minutes, blood pressure and heart rate were measured in the sitting position three times at 3–5 minute intervals with the appropriate size automated blood pressure cuff (Omron 5 series). Reported blood pressure is the mean of the two closest readings of both systolic and diastolic pressures. Waist circumference was measured one inch above the umbilicus. Bioelectrical impedance was used to measure BFP according to the protocol of the medical device (Bodystat 1500) (17).

#### Step 3: Biochemical measurements.

Finger-stick blood samples were obtained by a trained physician after a minimum 12-hour fast. Laboratory measurements of serum glucose and lipids were performed using the Cholestech LDX following the manufacturer’s procedure protocol. This finger-stick technique has been validated against venous blood sampling (18).

### Interviewers

During the design of the NCDS, an emphasis was placed on building local capacity. In furtherance of this philosophy, nine local, bilingual (Spanish and Tzu’tujil) community health workers were recruited and trained in the use of study protocols and also participated in a customized training and certification program in human subject research ethics.

### NCD risk factors

Hypertension was defined as systolic pressure ≥140 mmHg, diastolic pressure ≥90 mmHg, or being on medical therapy for hypertension. Overweight was defined as body mass index (BMI) of 25–29.9, and obese as BMI ≥30.0. By BFP, obesity was defined as ≥25% for men and ≥35% for women (19). Central obesity was waist circumference (WC) ≥80 cm for women and WC ≥90 cm for men, in accordance with the International Diabetes Federation (IDF) definition for Latinos. Stunting was defined as a height of <162 cm for men and <150 cm for women, which corresponds to two standard deviations below the median of the 2000 reference population in the United States of America (20). Diabetes was defined as fasting glucose ≥126 mg/dL or taking medication for known diabetes. Hypercholesterolemia was total cholesterol ≥200 mg/dL, hypertriglyceridemia was triglycerides ≥150 mg/dL, and low high-density lipoprotein (HDL) was HDL ≤40 mg/dL for men or HDL ≤50 mg/dL for women; being on medication to treat these conditions also met the definition. Metabolic syndrome was identified according to the IDF criteria (21). Ten-year risk for cardiovascular events was calculated using WHO risk charts (22).

Smoking and alcohol use were defined as any current use. Participants were considered sedentary if they reported less than 60 minutes of moderate to intense physical activity per week. Adequate fruit/vegetable intake was defined as at least five servings of fruits and vegetables per day.

### Data management and statistical analysis

Data entry was done using EpiData 2.1 and was subjected to systematic review for outlying data following the WHO STEPS protocol. Statistical analysis was conducted with SAS 9.4 (SAS Institute Inc., Cary, North Carolina, United States of America).

All survey responses were analyzed in accordance with the single-stage stratified random sampling design. The Santiago Atitlán community served as the stratification variable. Population and subpopulation parameter estimates for prevalence and averages were derived based on sampling inclusion probabilities (23) in which survey weights assigned to the respondents corresponded to age and gender demographic information from the 2005 Santiago Atitlán census. Variability in the population or subpopulation parameter estimates was ascertained via the Taylor series linear expansion technique (24).

Gender comparisons of continuous variables were conducted via linear regression weighted by probability of inclusion in the single-stage stratified random sample, while risk factor prevalences were compared between female and male subpopulations via similarly probability-weighted logistic regression. Association of NCD risk factors to literacy and education level was assessed via probability-weighted logistic regression. In these analyses, the NCD risk factors were treated as the response variables, while literacy and education were the predictor variables in age- and gender-adjusted analyses. Literacy was modeled as a binary variable and levels of education were modeled as ordinal categories. The Bonferroni correction was applied to correct for multiple comparisons.

### Ethical considerations

The study protocol conforms to the ethical guidelines of the 1975 Declaration of Helsinki and was approved by both partner institutions’ ethics committees: the University of Virginia’s Human Subjects’ Research Institutional Review Board (IRB) (Charlottesville, Virginia, United States of America) and the Institute of Nutrition of Central America and Panama (INCAP) IRB (Guatemala City, Guatemala). All participants gave verbal and written informed consent. All collected data were anonymized prior to analysis using linked participant identification numbers. Identifiable information was kept under lock and key at INCAP’s central office and was accessible only to the authors of the study.

## RESULTS

### Participants

Of the 501 persons screened, 350 agreed to participate, a response rate of 70.0%. Among these 350 participants, 308 subjects (88.0%) provided blood for biochemical analysis. Participants who provided blood did not differ from those who did not with respect to age, sex, or literacy (as a surrogate of socioeconomic status).

The mean age of the participants was 36.7 years old, and 72.3% were women. Educational attainment was low; 45.3% (95% confidence interval (CI): 38.9–51.8%) had no formal education, and an additional 23.7% (95% CI: 13.4–28.9%) did not complete primary school. Literacy was low at 54.2% (95% CI: 47.6–60.8%). Most men (83.1%; 95% CI: 72.3–93.0%) were self-employed, while most women (75.4%; 95% CI: 69.0–81.7%) were homemakers. Most participants (86.2%; 95% CI: 80.5–91.9%) reported earning less than 500 *quetzales* per month (∼US$ 65) (Table 1).

With respect to behavioral risk factors, prevalence of current smoking was 13.0% (95% CI: 5.4–20.6%) among men, while no women reported tobacco use. Similarly, no women reported alcohol use, but 19.6% (95% CI: 10.6%–28.6%) of men did. Most participants (75.0%; 95% CI: 68.7–81.2%) reported being sedentary, with <60 minutes of physical activity per week. The majority (91.6%; 95% CI: 88.4–94.8%) reported consuming less than the minimum recommended intake of fruits and vegetables (Table 2).

Anthropometric measures were notable for a high prevalence of stunting: 79.8% (95% CI: 73.8–85.8%). There was also a high rate of overweight and obesity, with a mean BMI of 27.0 (Table 3). Over one-third (36.7%; 95% CI: 29.5–42.6%) of participants were overweight (BMI 25–29.9), and an additional 25.2% (95% CI: 19.5–30.9%) were obese (BMI ≥30). This high rate of obesity was consistent with other measures of adiposity, including obesity by BFP (55.3%; 95% CI: 48.0–62.5%) and central obesity (45.1%; 95% CI: 38.1–52.1%) (Table 2). The gender difference with respect to obesity was large and statistically significant, with women far more likely to be obese than men as measured by BMI (37.3% vs. 12.4%, respectively; *P* < 0.05). This pattern held true for obesity by WC and BFP.

Hypertension was prevalent at 18.3% (95% CI: 13.1–23.6%), and rates did not differ significantly between the sexes (Table 2). Among hypertensive participants, the diagnosis was new to over three-quarters of them.

With respect to biochemical measurements, hypertriglyceridemia was highly prevalent at 64.0% (95% CI: 56.9–71.1%), while only 11.9% (95% CI: 7.4–16.5%) had a total cholesterol level higher than 200 mg/dL. Most of the group had low HDL levels (82.7%). None of the dyslipidemic patients had previously been diagnosed. In contrast, the rate of diabetes (fasting glucose ≥126 mg/dL) was only 3.0% (95% CI: 1.1–4.8%). Of those with diabetes, less than half knew they had elevated glucose. There was no statistically significant difference in the prevalence of any of the biochemical NCD risk factors between the sexes.

**TABLE 1.** Demographic characteristics, by sex, in the Noncommunicable Disease Surveillance (NCDS) study population in Santiago Atitlán, Guatemala, 2012–2013

Variable	Category	Overall *n* = 336^a^ Estimate [95% CI^b^]	Men *n* = 92 Estimate [95% CI]	Women *n* = 243 Estimate [95% CI]
Mean age (yrs)		36.7 [35.0, 38.4]	36.6 [33.6, 39.6]	36.8 [34.9, 38.7]
Education (%)	No formal schooling	45.3 [38.9, 51.8]	35.9 [25.3, 46.5]	54.1 [46.7, 61.4]
	Some primary school	23.7 [13.4, 28.9]	23.9 [14.7, 33.1]	25.3 [17.7, 29.2]
	Completed primary school	15.8 [9.9, 21.6]	22.0 [11.4, 32.6]	10.0 [4.7, 15.2]
	Completed secondary school or higher	15.2 [9.5, 20.9]	18.2 [8.2 28.1]	12.5 [6.5, 18.5]
Literacy (%)	Literate	54.2 [47.6, 60.8]	65.0 [54.2, 60.8]	44.0 [36.5, 51.5]
Ethnic group (%)	Tzu’tujil	97.0 [94.2, 100]	96.0 [90.4, 100]	98.0 [96.3, 99.7]
	Kaqchikel	1.1 [0.0, 2.6]	1.5 [0.0, 4.4]	0.7 [0.0, 1.6]
	Ladino	1.9 [0.0, 4.3]	2.5 [0.0, 7.4]	1.2 [0.0, 2.7]
Marital status (%)	Single	20.1 [13.6, 26.6]	18.9 [8.0, 29.7]	21.3 [13.9, 28.6]
	Married	61.8 [54.9, 68.7]	66.4 [54.7, 28.2]	57.5 [49.8, 65.1]
	Separated	15.5 [10.9, 20.0]	12.4 [5.0, 19.8]	18.3 [12.9, 23.7]
	Divorced	0.2 [0.0, 0.4]	0.0	0.3 [0.0, 0.7]
	Widowed	2.4 [0.9, 4.0]	2.3 [0.0, 4.9]	2.7 [0.9, 4.4]
Occupation (%)	Employed, government	1.6 [0.0, 3.8]	2.8 [0.0, 7.3]	0.5 [0.0, 1.4]
	Employed, private sector	0.8 [0.0, 1.7]	1.0 [0.0, 2.4]	0.6 [0.0, 1.8]
	Self-employed	51.6 [44.9, 58.4]	83.1 [73.2, 93.0]	22.7 [16.5, 28.9]
	Unpaid work (volunteer)	1.8 [0.0, 3.8]	3.7 [0.0, 8.0]	0.0
	Student	1.5 [0.0, 3.9]	3.1 [0.0, 8.1]	0.0
	Stay-at-home	39.3 [33.3, 45.2]	0.0	75.4 [69.0, 81.7]
	Retired	0.0	0.0	0.0
	Unemployed	3.5 [0.0, 7.0]	6.3 [0.0, 13.4]	0.9 [0.0, 2.6]
Income (in quetzales (Q); approx. 8Q = US$ 1) (%)	500Q or less per month	86.2 [80.5, 91.9]	79.5 [69.3, 89.7]	93.6 [89.8, 97.3]

***Data source:*** NCDS Study.

a Number of subjects with complete demographic data for inclusion in this table.

b CI = confidence interval.

The prevalence of metabolic syndrome was 36.0% (95% CI: 29.2–42.8%); it was more common in women (56.7%; 95% CI: 48.4–65.0%) than in men (14.7%; 95% CI: 6.2–23.3%) (*P* < 0.05). Despite this finding, most of the population was classified as having a low 10-year risk of a major cardiovascular event, with only 2.8% of individuals over age 40 classified as intermediate-to-high risk (the WHO risk score tables only apply to people above 40 years of age).

There was a strong inverse association between obesity by BFP and both literacy and level of education. The odd ratios were 0.33 (95% CI: 0.18–0.64, *P* < 0.05) and 0.52 (95% CI: 0.37–0.72, *P* < 0.001), respectively. In other words, the more highly educated the individual, the lower the likelihood that he or she was obese. No other statistically significant associations were found between literacy or level of education and NCD risk factors (Table 4).

Participant knowledge of NCDs was low, with only one-fifth of participants (20.1%) answering at least seven of nine simple questions about NCDs correctly (Table 5).

## DISCUSSION

This study joins a small body of research examining noncommunicable disease epidemiology in indigenous Latin American populations. It identified a surprisingly high prevalence of numerous risk factors, including obesity in women, hypertension, hypertriglyceridemia, and metabolic syndrome.

The prevalence of obesity of 37.3% among women in this study was much higher than the 25.8% found in a cohort of nonindigenous, urban Guatemalans living in Villa Nueva (25), and it approaches the 43% obesity rate among Latino women living in the United States of America (26). On the other hand, men in this study were less obese (12.4%) than both those in Villa Nueva (16.0%) and the United States cohort (37%). The prevalence of central obesity was even more striking than obesity by BMI. Over three-quarters of the women in this study had central obesity, which is higher than the 68.3% prevalence among women in Villa Nueva. This is a particularly notable addition to the literature, since it was expected that women in a rural community such as Santiago would have lower rates of obesity than their urban counterparts. This study was not designed to be able to address directly the question of why women’s rates of obesity were so much higher than men’s, but the explanation may relate to the differing lifestyles of men and women in Santiago. Men typically engage in physically intensive labor, while women are more likely to work at home, in markets, or in other occupations requiring less physical activity. Alternatively, it is possible that genetic influences predispose these women to central obesity. Further research is needed to explore these mechanisms and to determine whether the high rate of obesity increases cardiovascular and diabetes risk, as it does in other ethnic groups.

**TABLE 2.** Prevalence and 95% confidence intervals (CI) of anthropometric and biochemical noncommunicable disease risk factors, by sex, in the Noncommunicable Disease Surveillance study population in Santiago Atitlán, Guatemala, 2012–2013

Variable		Overall (*n* = 296^a^) % [95% CI]	Men (*n* = 83) % [95% CI]	Women (*n* = 213) % [95% CI]
Anthropometric risk factors^b^				
Body mass index (BMI)	Normal^c^	38.6 [31.2, 46.0]	57.5 [45.5, 69.8]	20.8 [14.5, 27.1]
	Overweight^c^	36.7 [29.5, 42.6]	30.1 [19.1, 41.1]	41.9 [34.2, 49.6]
	Obese^c^	25.2 [19.5, 30.9]	12.4 [5.3, 19.6]	37.3 [29.8, 44.8]
Central obesity^c^		45.1 [38.1, 52.1]	14.8 [6.5, 23.1]	73.6 [66.3, 80.3]
Obesity by percentage body fat (BFP)^c^		55.3 [48.0, 62.5]	35.7 [24.5, 46.9]	73.8 [66.5, 81.0]
Stunted		79.8 [73.8, 85.8]	76.7 [66.1, 87.3]	82.7 [76.9, 88.6]
Hypertension		18.3 [13.1, 23.6]	20.0 [10.9, 29.2]	16.8 [11.5, 22.1]
Known hypertension		3.3 [0.64, 6.1]	4.3 [0.0, 9.5]	2.4 [0.5, 4.4]
Previously unknown hypertension		15.0 [10.3, 19.7]	15.7 [7.6, 23.9]	14.3 [9.3, 19.4]
Biochemical risk factors^b^				
Diabetes mellitus (DM)		3.0 [1.1, 4.8]	1.3 [0.0, 3.2]	4.6 [1.6, 7.7]
Known DM		1.3 [0.1, 2.5]	1.3 [0.0, 3.2]	1.4 [0.0, 2.9]
Unknown DM		1.7 [0.3, 3.0]		3.2 [0.5, 5.9]
Hypercholesterolemia		11.9 [7.4, 16.5]	9.7 [2.6, 16.8]	14.1 [8.4, 19.7]
Low HDL cholesterol		82.7 [76.9, 88.5]	78.0 [67.5, 88.5]	87.2 [81.8, 92.7]
Elevated LDL		5.0 [2.2, 7.8]	3.9 [0.0, 7.8]	6.0 [2.0, 9.9]
Hypertriglyceridemia		64.0 [56.9, 71.1]	65.1 [53.0, 77.2]	62.9 [55.3, 70.5]
Metabolic syndrome^c^		36.0 [29.2, 42.8]	14.7 [6.2, 23.3]	56.7 [48.4, 65.0]
Behavioral risk factors				
Current tobacco use		6.3 [2.5, 10.0]	13.0 [5.4, 20.6]	0.0
Alcohol within last 12 months		9.4 [4.9, 13.9]	19.6 [10.6, 28.6]	0.0
<5 Servings of fruits and vegetables per day		91.6 [88.4, 94.8]	95.0 [90.5, 99.5]	88.4 [83.7, 93.0]
Sedentary***^c^***		75.0 [68.7, 81.2]	64.5 [53.3, 75.7]	84.7 [79.2, 90.3]

***Data Source:*** NCDS Study.

a Number of subjects with complete anthropometric and biochemical data.

b Overweight: BMI 25-29.9; Obese: BMI ≥30; Central obesity: Waist circumference ≥90 cm in men or ≥80 cm in women; Obesity by BFP: ≥25% body fat in men or ≥35% body fat in women; Stunted: Height ≤162 cm in men or ≤150 cm in women; Hypertension: Systolic ≥140 mmHg or diastolic ≥90 mmHg; Diabetes: Fasting glucose ≥126 mg/dL; Hypercholesterolemia: Total cholesterol ≥200 mg/dL; Low HDL: HDL ≤40 mg/dL in men or ≤50 mg/dL in women; High LDL: LDL ≥130 mg/dL; Hypertriglyceridemia: Triglycerides ≥150 mg/dL; Metabolic syndrome by IDF criteria; Sedentary: <60 min of moderate-intense physical activity per week.

c *P* < 0.05 for difference between men and women by logistic regression.

Obesity was not the only NCD risk factor found to be markedly higher than expected. Over three-quarters of the study participants were dyslipidemic. However, unlike for obesity, there was no significant difference in prevalence between the sexes. Once again, the Santiago population was found to have a higher rate of dyslipidemias than their urban, nonindigenous counterparts in Villa Nueva. For instance, hypertriglyceridemia was present in 53.2% of Villa Nueva participants (25) compared to 64.0% in our indigenous population. Of interest is the difference in triglyceride levels between our study population and Latinos in the United States of America. Mean triglycerides in women and men were 209.7 mg/dL and 221.5 mg/dL, respectively, in this study versus 119.9 mg/dL and 149.0 mg/dL, respectively, among U.S. Latinos (27). Possible explanations of this high rate of hypertriglyceridemia include genetic predisposition and high intake of carbohydrates, including refined carbohydrates. More research is needed to explore the reasons for these differences.

The finding that the prevalence of major NCD risk factors in this rural indigenous community in a LMIC approaches that in the United States of America is a cause for concern and represents a public health threat for Guatemala. Indigenous communities constitute 40% of the Guatemalan population and are the most impoverished and underserved groups in the country. Thus, systematic and aggressive prevention efforts are needed alongside initiatives to increase availability of therapies for established NCDs.

The 18.3% prevalence of hypertension in this study’s participants was similar to that reported in their urban, nonindigenous Guatemalan counterparts in Villa Nueva (17%) but less than the 24.4% hypertension prevalence found among U.S. Latinos. It was surprising to find such a low rate of diabetes in our study, given the high degree of overweight and obesity. The prevalence of diabetes is also discordant with the prevalence of other NCD risk factors (25–27). One possible explanation is that this study may have captured the population at a very early stage in the epidemiologic transition. A small community-based study of 49 women from Santiago Atitlán in 2007 (28) found rates of overweight and obesity of 22.4% and 24.5%, respectively. Those rates are notably lower than the ones found in this study five to six years later, although different sampling methods make direct comparison difficult. Nevertheless, the difference suggests the possibility that the prevalence of obesity has increased rapidly over the last decade and supports the theory that insufficient time has passed to see the metabolic effect of obesity leading to diabetes. An alternative explanation that merits consideration is that standard cut-offs for obesity and central obesity derived from non-Mayan populations may not accurately predict risk in this indigenous group. It has been well established that optimal BMI cut points can vary by population (29). For instance, there is variation in suggested BMI cut points among and between Asian populations (30), with obesity thresholds ranging from 26 to 31. One study examined BMI and WC risk thresholds among nonindigenous Guatemalan adults and suggested that optimal risk category cut-offs may differ from international norms (20). Determination of the optimal BMI and WC cut-offs for predicting NCD risk in this and other ethnic groups remains an important area for future research.

No significant association was found between literacy or level of education and 15 out of 16 NCD risk factors after correcting for multiple comparisons (Table 4). This lack of consistent association between these two surrogates of socioeconomic status and NCD risk factors is consistent with other studies in LMICs (31). Poor communities in early epidemiologic transition exhibit heterogeneous behavior in relation to emergence of NCD risk factors. The subgroups with higher purchasing capacity may become early adopters of lifestyles that promote NCD risk (31). Santiago may represent a community with mixed low- and middle-income families whose lifestyles produce a differential effect.

**TABLE 3.** Values for anthropometric and biochemical risk factors and 95% confidence intervals (CI), by sex, in the Noncommunicable Disease Surveillance (NCDS) study population in Santiago Atitlán, Guatemala, 2012–2013

Variable	Units	Overall *n* = 296^a^ Mean [95% CI]	Men *n* = 83 Mean [95% CI]	Women *n* = 213 Mean [95% CI]
Anthropometric risk factors				
BMI*^b^*		27.0 [26.3, 27.6]	25.3 [24.3, 26.3]	28.5 [27.9, 29.2]
Waist circumference*^b^*	cm	83.7 [82.4, 85.1]	81.7 [79.1, 83.7]	85.6 [83.9, 87.4]
Body fat percentage*^b^*	%	30.8 [29.4, 32.3]	22.4 [21.1, 23.8]	38.8 [37.8, 39.8]
Height*^b^*	cm	151.5 [150.2, 152.8]	157.9 [156.1, 159.7]	145.4 [144.5, 146.3]
Blood pressure, systolic	mmHg	122.2 [120.0, 124.5]	123.1 [119.3, 127.0]	121.4 [118.9, 123.9]
Blood pressure, diastolic	mmHg	75.6 [74.2, 77.1]	76.1 [73.7, 78.5]	75.2 [73.4, 74.0]
Biochemical risk factors				
Total cholesterol	mg/dL	162.8 [158.1, 167.5]	160.4 [152.8, 168.0]	165.0 [159.6, 170.5]
Triglycerides	mg/dL	215.4 [197.9, 232.9]	221.5 [194.0, 249.0]	209.7 [187.8, 231.5]
HDL	mg/dL	36.5 [34.5, 38.6]	34.9 [31.3, 38.5]	38.1 [36.0, 40.1]
LDL	mg/dL	87.6 [84.2, 91.0]	89.0 [83.9, 94.0]	86.3 [81.6, 91.0]

***Data source:*** NCDS Study.

a Number of subjects with complete anthropometric and biochemical data.

b *P* < 0.05 for difference between men and women by linear regression.

**TABLE 4.** Odds ratios and 95% confidence intervals (CI) of association between noncommunicable disease risk factors and literacy or level of education, sex-adjusted, in the Noncommunicable Disease Surveillance (NDCS) study population in Santiago Atitlán, Guatemala, 2012–2013

Risk factor	Literate (Yes : No)		Level of education (One category increase in education)^b^	
	Adjusted odds ratio [95% CI]	*P*-value^a^ (uncorrected *P*)	Adjusted odds ratio [95% CI]	*P*-value (uncorrected *P*)
BMI ≥25	0.80 [0.41, 1.55]	1.000 (0.509)	0.75 [0.56, 1.00]	1.000 (0.345)
BMI ≥30	0.74 [0.40, 1.38]	1.000 (0.345)	0.83 [0.62, 1.11]	1.000 (0.204)
Central obesity	0.66 [0.33, 1.34]	1.000 (0.252)	0.75 [0.54, 1.03]	1.000 (0.074)
Obesity by body fat percentage	0.33 [0.18, 0.64]	0.016 (0.001)	0.52 [0.37, 0.72]	<0.001 (<0.001)
Stunted	0.82 [028, 1.37]	1.000 (0.239)	0.82 [0.57, 1.21]	1.000 (0.331)
Hypertension	0.67 [0.37, 1.20]	1.000 (0.177)	0.72 [0.52, 0.99]	0.688 (0.043)
Diabetes mellitus	0.26 [0.07, 1.00]	0.801 (0.050)	0.43 [0.16, 1.14]	1.000 (0.090)
Hypercholesterolemia	0.72 [0.30, 1.74]	1.000 (0.463)	0.95 [0.64, 1.41]	1.000 (0.808)
Low HDL cholesterol	0.93 [0.40, 2.17]	1.000 (0.817)	0.87 [0.59, 1.28]	1.000 (0.479)
Elevated LDL	0.34 [0.10, 1.10]	1.000 (0.073)	0.78 [0.42, 1.46]	1.000 (0.437)
Hypertriglyceridemia	0.61 [0.32, 1.15]	1.000 (0.126)	0.65 [0.48, 0.87]	0.064 (0.004)
Metabolic syndrome	0.68 [0.35, 1.33]	1.000 (0.265)	0.72 [0.53, 0.99]	0.720 (0.045)
Tobacco use^c^	0.31 [0.07, 1.30]	1.000 (0.109)	0.31 [0.14, 0.74]	0.128 (0.008)
Alcohol use in last 12 months^c^	1.17 [0.41, 3.35]	1.000 (0.774)	0.70 [0.43, 1.12]	1.000 (0.137)
<5 Servings of fruits and vegetables per day	1.48 [0.63, 3.52]	1.000 (0.370)	1.53 [0.61, 3.86]	1.000 (0.362)
Sedentary	0.48 [0.23, 0.98]	0.720 (0.045)	0.79 [0.50, 1.25]	1.000 (0.318)

***Data source:*** NCDS Study.

a *P*-values (α = 0.05) were determined by logistic regression with Bonferroni correction for multiple comparisons.

b Ordinal categories were: No formal education, Some primary education, Complete primary education, Complete secondary school or higher.

c No gender adjustment due to females having zero prevalence for the risk factor.

This study also revealed that adults in Santiago had a poor understanding of NCDs, with many respondents being unable to answer questions on relatively basic NCD-related concepts. For example, only one-fifth of individuals were able to correctly answer a multiple choice question that asked them to describe a classic heart attack. This suggests that basic cardiovascular health education is a potentially low-cost, easy-to-implement intervention through which to begin tackling NCDs.

Comparison between the reported prevalence of NCD risk factors in the Santiago population and rates in nonindigenous Guatemalans and U.S. Latinos highlights that the indigenous population of Santiago Atitlán has NCD risk factor prevalences similar to communities further along in the epidemiologic transition. Indigenous communities in Guatemala share many cultural features and experience similar socioeconomic hardships and lack of adequate health care. Thus, it can be speculated that the findings of this study are likely generalizable to other indigenous communities in the country.

**TABLE 5.** Knowledge of noncommunicable diseases and their risk factors, as measured by number of questions answered correctly, among participants (n = 336) in the Noncommunicable Disease Surveillance (NCDS) study population in Santiago Atitlán, Guatemala, 2012–2013

Number of questions answered correctly	Percent answering correctly [95% CI]	Cumulative percent
0 / 9	0.7 [0.0, 1.6]	0.7
1 / 9	3.3 [1.6, 5.0]	4.0
2 / 9	7.5 [4.0, 11.0]	11.6
3 / 9	10.9 [7.1,14.7]	22.5
4 / 9	19.8 [14.3, 25.4]	42.3
5 / 9	19.1 [14.1, 24.1]	61.4
6 / 9	18.5 [12.8, 24.3]	79.9
7 / 9	13.0 [7.9, 18.1]	92.9
8 / 9	4.6 [1.4, 7.7]	97.5
9 / 9	2.5 [0.0, 5.1]	100.0

***Data source:*** NCDS Study.

We found it challenging to work in a low-resource setting with major cultural barriers—a common hurdle in global health research. By applying a community-based, participatory approach that emphasized best practices and partnership with local organizations, we obtained a satisfactory response rate of 70%. Furthermore, this approach enabled interviews to be conducted in the participants’ native language, minimizing the risk of translation errors. Beyond the results of this study, bringing community groups together may have a lasting impact on NCD prevention efforts in the area. This approach contributed to local capacity building and helped establish connections for future collaboration.

### Limitations

This population-based study utilized home-based surveys, limiting the sample to individuals at home during the day. The cultural role of women in the community and an unbalanced (i.e., lower) participation of women in the work force likely contributed to the over-representation of women in our sample (72.6%). An attempt was made to correct for participation bias by applying multi-strata sampling weights to the analysis, but the risk of residual confounding or bias still exists. The 30% of screened individuals who did not respond and the additional 12% who did not complete biochemical measurements may have differed from those who participated, potentially introducing selection bias into the data. Thus, results must be interpreted with caution in the context of these limitations.

### Conclusions

This community-based, participatory, cross-sectional study of noncommunicable disease risk factors in a rural Mayan community in a LMIC demonstrated that this community suffers from high rates of certain NCD risk factors. Specifically, high prevalences of obesity, hypertension, dyslipidemias, and metabolic syndrome were found, underscoring the need for effective, low-cost interventions to reduce the risk of noncommunicable diseases in this community and others like it.

It is likely that the high prevalence of risk factors will lead to rising rates of diabetes mellitus and cardiovascular disease in this community. It is important to confirm that similar trends are occurring in other indigenous populations in Guatemala to determine the generalizability of these findings for the purpose of applying common preventive efforts. In addition to actions aimed at improving health literacy relative to NCDs, systematic and cost-effective efforts for the detection of individuals with NCD risk factors are needed. Multi-institutional efforts to train health care personnel in NCD prevention and improve access to essential drugs would also be of great benefit in curtailing this emerging public health threat.

#### Acknowledgements.

We are in debt to the community of Santiago Atitlán, Guatemala for allowing us to come into their community for this investigation. In particular, we would like to thank the community health workers and other local leaders that contributed to the success of this project: Leticia Ixcaya, Odilia Ixcaya, Marcos Sicay, Santiago Sicay, Ana Maritza Lacam, Josefa Sicay, Josefa Damián, Lucrecia Barán, and Marilu Quievac. We hope that this study contributes to the health of this community and others.

#### Disclaimer.

Authors hold sole responsibility for the views expressed in the manuscript, which may not necessarily reflect the opinion or policy of the *RPSP/PAJPH* or PAHO.
